# Ion Channel Genes in Painful Neuropathies

**DOI:** 10.3390/biomedicines11102680

**Published:** 2023-09-29

**Authors:** Milena Ślęczkowska, Kaalindi Misra, Silvia Santoro, Monique M. Gerrits, Janneke G. J. Hoeijmakers

**Affiliations:** 1 Department of Toxicogenomics, Maastricht University, 6229 ER Maastricht, The Netherlands; milena.molasy@maastrichtuniversity.nl; 2 Department of Neurology, School of Mental Health and Neuroscience, Maastricht University Medical Centre+, 6229 ER Maastricht, The Netherlands; 3 Laboratory of Human Genetics of Neurological Disorders, IRCCS San Raffaele Scientific Institute, INSPE, 20132 Milan, Italy; misra.kaalindi@hsr.it (K.M.); santoro.silvia@hsr.it (S.S.); 4 Department of Clinical Genetics, Maastricht University Medical Centre+, 6229 HX Maastricht, The Netherlands; monique.gerrits@mumc.nl

**Keywords:** neuropathic pain, ion channel genes, neuropathy, variants, pathophysiology, channelopathies

## Abstract

Neuropathic pain (NP) is a typical symptom of peripheral nerve disorders, including painful neuropathy. The biological mechanisms that control ion channels are important for many cell activities and are also therapeutic targets. Disruption of the cellular mechanisms that govern ion channel activity can contribute to pain pathophysiology. The voltage-gated sodium channel (VGSC) is the most researched ion channel in terms of NP; however, VGSC impairment is detected in only <20% of painful neuropathy patients. Here, we discuss the potential role of the other peripheral ion channels involved in sensory signaling (transient receptor potential cation channels), neuronal excitation regulation (potassium channels), involuntary action potential generation (hyperpolarization-activated cyclic nucleotide-gated channels), thermal pain (anoctamins), pH modulation (acid sensing ion channels), and neurotransmitter release (calcium channels) related to pain and their prospective role as therapeutic targets for painful neuropathy.

## 1. Introduction

Peripheral Neuropathy (PN) is a debilitating illness caused by peripheral nerve damage. Depending on the kind of nerve implicated, this condition impairs sensation, movement, pain transmission, and gland or organ function. Over 50% of individuals experiencing PN report pain symptoms due to a lesion or disease in their somatosensory system [1,2]. As a result, these individuals are classified as Painful PN (PPN) patients, and the pain experience can significantly impact those affected. The current pharmacological treatment of PPN is limited by side effects and often only has moderate efficacy [2]. Despite recent advances in pain research, the pathophysiology of PPN remains largely unknown, hindering the development of novel therapeutic drugs [3].

**Figure 1:**
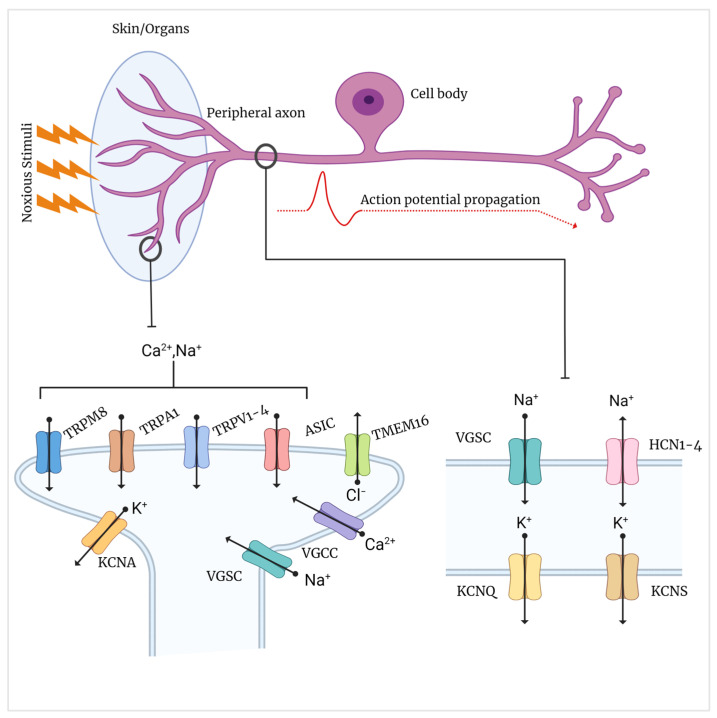
The activation of ion channels at the peripheral terminals in response to unpleasant stimuli causes membrane depolarization and the generation of action potentials in afferent fibers. These action potentials are generated by ion channel activation and propagate along axons to postsynaptic nerve terminals in the spinal dorsal horn. TMEM16 Transmembrane protein 16 (Anoctamins), TRPV Transient Receptor Potential Vanilloid, TRPA1 Transient Receptor Potential Ankyrin 1, TRPM8 Transient Receptor Potential Melastatin 8, ASIC Acid-Sensing Ion Channel, VGCC Voltage-dependent Calcium Channel, VGKC Voltage-Gated Potassium Channel, VGSC Voltage-Gated Sodium Channel, HCN Hyperpolarization-activated, and Cyclic Nucleotide-gated channel [10,12,13].

It is important to acknowledge that there is a correlation between pain perception and genetic variants in Ion Channel Genes (ICGs) [4]. As these ion channels play a critical role in generating, transmitting, and transforming nerve signals in the peripheral nerves, they must be thoroughly examined as promising molecular targets for PPN therapy [5]. Voltage-Gated Sodium Channels (VGSCs) are the most researched ICGs and have been linked to disorders such as congenital insensitivity to pain (CIP), paroxysmal extreme pain disorder (PEPD), erythromelalgia, small fiber neuropathy (SFN), and painful diabetic peripheral neuropathy (PDPN) [6]. While VGSCs play a crucial role in neuronal excitability and pain processing, alterations in these genes can only account for a portion of the complexity of PPN [7]. Recent studies have shown that other ICGs are involved in pain signaling, modulation, and transmission (Figure 1) [5,8,9]. This review delves into the growing function of ICGs in PPN, aside from VGSCs, which have been previously extensively discussed in recent reviews [7,10,11]. Specifically, we focus on ion channels that have a known connection to pain and/or peripheral neuropathy, such as calcium and potassium channels, hyperpolarization-activated cyclic nucleotide-gated channels (HCN), anoctamins (TMEM16/ANO), transient receptor potential cation channels (TRP), and acid-sensing ion channels (ASIC).

## 2. Role of Ion Channels in Painful Peripheral Neuropathy

### 2.1. Voltage-Gated Ca^2+^ Channels and Calcium Signaling

Voltage-gated calcium channels (VGCCs) in excitable cells help neurons communicate by converting action potentials into calcium ion flows, triggering the release of neurotransmitters. VGCCs consist of a pore-forming α_1_ subunit (Ca_v_1-3.*x*) associated with multiple auxiliary subunits (α_2_δ, β, and γ), except Cav3.*x*, which forms T-type Ca^2+^ channel without any auxiliary subunits. Moreover, the γ subunit is only present in calcium channel complexes in skeletal muscle [14]. The α_1_ subunit of VGCC creates the pore that facilitates the influx of calcium ions, whereas the auxiliary subunits are responsible for membrane trafficking, expression, and biophysical property regulation [15,16]. VGCCs are classified into L-, N-, P/Q-, R-, and T-types depending on the voltage required for their activation and biochemical properties. VGCCs in the sensory system can cause pain when their function is disrupted. N-type and T-type channels are studied for pain relief, but the roles of other channels are not well known. Several studies, including both animal and clinical studies, have demonstrated that there is a connection between PPN and VGCCs of the N- (*CACNA1B*), P/Q- (*CACNA1A*), R-(*CACNA1E*), and T-type (*CACNA1G*, *CACNA1H,* and *CACNA1I*) and auxiliary subunits (*CACNA2D1*, *CACNA2D2*, *CACNA2D3*, and *CACNA2D4*) (Table 1).

#### 2.1.1. CACNA1A and CACNA1B

The Ca_v_2.1 (P/Q-type) and Ca_v_2.2 (N-type) channels, encoded by *CACNA1A* and *CACNA1B*, are expressed at the presynaptic terminals of dorsal root ganglion (DRG) neurons. Ca_v_2.1 regulates excitatory neurotransmitter release and somatodendritic cell neural excitability [14,15]. This VGCC was the first to be genetically linked with complex multi-genic disorders like epilepsy, migraine, and ataxia. Mutations in the *CACNA1A* gene can lead to different conditions. Gain-Of-Function (GOF) mutations are associated with familial hemiplegic migraine 1, while Loss-Of-Function (LOF) mutations have been linked to episodic ataxia type 2. The GOF mutations in this gene have been found to alter channel characteristics and synaptic transmission in the pain pathway [33]. Ca_v_2.1 α_1_ null mutant mice demonstrated pro-nociceptive responses to inflammatory and Neuropathic Pain (NP) models but anti-nociceptive responses to noxious heat stimuli. In a separate study, mice with a spontaneous mutation in the Ca_v_2.1 channel, which reduced the activation voltage sensitivity, showed hypoalgesic responses to heat, mechanical, and chemical stimuli [34]. Recently, a GOF mutation in the *CACNA1A* gene has been linked to trigeminal neuralgia, as mentioned in Table 1. This mutation has been observed to affect channel gate kinetics, indicating that alterations in Ca_v_2.1-dependent synaptic transmission in the trigeminal system may contribute to the processing of pain [17].

Ca_v_2.2 (N-type) VGCCs are present in the dendritic shafts and presynaptic terminals of neurons in both the central and peripheral nervous systems. They are responsible for transmitting nociceptive signals in the spinal cord’s dorsal horn from A-δ and C nerve fibers [34]. Several studies have demonstrated that blocking or deleting Ca_v_2.2 channels can alleviate pain. According to research on rodent pain models or in vitro, ω-conotoxins from marine cone snails and spider venom can block Ca_v_2.2 channels [35]. Out of these tested animal models, only ω-conotoxin (MVIIA or SNX-111) from *Conus magus* has been clinically approved under the name of Ziconotide (Prialt^®^) for chronic pain administered intrathecally and is known to have severe neurological and psychiatric side effects [34]. It was recently shown that these channels found in epidermal nerve terminals only play a role in heat sensitivity following nerve damage, not mechanical sensitivity. Because heat sensitivity is a common symptom of capsacin-induced pain, intraplantar ziconotide would lead to decreased heat sensitivity in such pain [36]. Another recent study involving *cacna1b* gene knockin mouse with the hemagglutinin tag, post partial sciatic nerve ligation, exhibited an increased expression of the Ca_v_2.2 channel in medium/large DRG neurons with a coexpression of a glial cell line-derived neurotrophic factor (GDNF) family ligand receptor (GFR α1) located in low-threshold mechanoreceptors, and the increased expression of the Ca_v_2.2 channel was dependent on the auxiliary subunit complex α2δ-1; however, the expression of GFR α1 was not. This led to increased Ca_v_2.2 channel trafficking to mechanoreceptor terminals, indicating increased neurotransmission [37]. Moreover, the Ca_v_2.2 channel forms complexes with µ-opioid receptors and morphine; a µ-opioid receptor agonist inhibits this channel, providing pain relief. However, the effectiveness of morphine is reduced by the alternative splicing of the Ca_v_2.2 channel at exons 37a and 37b, as this changes the formation of the channel complex. It seems that a possible solution to this issue is a spider peptide called “Phα1β”. This peptide can block both TRPA1 and Ca_v_2.2 channels in postoperative mice who have received varying doses of morphine [38]. Regarding the genetic markers in this channel, LOF variants in the Ca_v_2.2 channel have been linked to progressive epilepsy dyskinesia, while GOF variants were once thought to be linked with myoclonic dystonia with painful cramps, but this has since been discredited. LOF mutations reduce neurotransmission by affecting Ca^2+^ influx, but the mechanism behind GOF variations is not fully understood [39,40,41]. A rare genetic variant in the *CACNA1B* gene, which encodes the Ca_v_2.2 channel, was found in patients with post-operative pain and high morphine use; however, its impact on the protein requires further investigation for proper phenotypic association [42].

#### 2.1.2. CACNA1G/1H/1I

Neuronal T-type calcium channels Ca_v_3.1-3 are encoded by *CACNA1G*, *CACNA1H,* and *CACNA1I*, respectively, and found on the cell bodies and dendrites of neurons. Regarding NP, knockout mice models studies for Ca_v_3.1 and Ca_v_3.3 have revealed their association with trigeminal NP, while the former channel was also associated with peripheral pain [43,44]. On the other hand, the expression of Ca_v_3.2 channels in the lamina I and II of mouse spinal cord and electrophysiological studies have shown their importance in sensory signal processing at the dorsal horn. Moreover, the Ca_v_3.2^GFP-Flox^ KI mouse model revealed Ca_v_3.2’s role in chronic pain and sensory processing. It affects subthreshold and suprathreshold properties in spinal cord neurons; modulates synaptic transmission; and influences action potential features, firing patterns, and spike coding. This unique role has implications for pain pathways and therapeutic potential [43]. In paclitaxel-treated rats, Ca_v_3.2 channels were co-localized with Toll-like receptor 4, and inhibiting either gene prevented paclitaxel-induced neuropathy [45]. Comparatively, intrathecal Ca_v_3.2 anti-sense oligonucleotides generated a reduction in T-type currents in DRG neurons, causing a decrease in nociceptive responses in naïve and neuropathic rats, while targeting Ca_v_3.1 or Ca_v_3.3 had no impact [46]. Medicinal chemistry has led to potential T-type calcium channel blockers for pain, but clinical success is uncertain [47]. Ca_v_3.2’s role in pain and its regulation through post-translational modifications present avenues for therapeutic development. Selective inhibitors like 5bk and betulinic acid show promise in neuropathy rodent models, highlighting the potential for targeted pain relief [48]. In a case of pediatric chronic pain, two heterozygous missense variants in the *CACNA1H* gene were discovered, but their functional impact on Ca_v_3.2 channels remains uncertain due to variable experimental outcomes [43]. Recently, a rare *CACNA1H* point mutation (Table 1) was demonstrated to segregate in a family with writer’s cramp and was also observed in a patient with bilateral trigeminal neuralgia. This point mutation is predicted to affect the different isoforms of the *CACNA1H* gene (±exon 26) and influences the current behavior of this channel. Therefore, it is pertinent to study the effect of variants in this channel with respect to different isoforms of the *CACNA1H* gene [18,49]. Other known variants in the *CACNA1H* gene with a possible association with corneal neuralgia are reported in Table 1 [24].

#### 2.1.3. CACNA2D1/D2/D3/D4

The auxiliary subunits α_2_δ, β, and γ regulate the biophysical characteristics and trafficking of the α_1_ subunit, whereas the α_2_ subunit forms a complex with the δ subunit. These subunits, which are separated into complexes α_2_δ-1 to α_2_δ-4, are encoded by the following genes: *CACNA2D1*, *CACNA2D2*, *CACNA2D3*, and *CACNA2D4* [14].

The auxiliary subunit α_2_δ -1 plays a crucial role in calcium channel trafficking and is linked to pain pathways. Following neuropathic injury, α_2_δ-1 is upregulated, and its knockout delays neuropathic hypersensitivity development. This subunit is expressed in DRG neurons, particularly in small neurons, and influences the distribution of Ca_v_2.2 channels, while global α_2_δ-1 ablation demonstrates that α_2_δ-1 is vital for directing Ca_v_2.2 channels to the cell surface and presynaptic terminals in DRG neurons and the dorsal horn of the spinal cord. Despite disruptions in Ca_v_2.2 localization, there were no significant reductions in other presynaptic markers or postsynaptic markers. Meanwhile, synapse density remained relatively unchanged, and the intensity of Ca_v_2.2 in puncta clusters was markedly reduced without α_2_δ-1. Further exploration through the use of techniques such as electron microscopy is necessary to understand the potential changes in synaptic morphology that are caused by α_2_δ-1 ablation [50]. After nerve injury, α_2_δ subunit expression increases in peripheral DRG and spinal cord neurons, making α_2_δ a therapeutic target for gabapentinoids like gabapentin, pregabalin, and mirogabalin. These drugs, which are developed for epilepsy, also bind to α_2_δ-1 and α_2_δ-2 subunits, reducing calcium influx and neurotransmission. While mainly used for epilepsy, gabapentinoids effectively alleviate seizures and are approved for such treatment [51].

The newfound role of α_2_δ in connection with thrombospondins (TSPs), especially thrombospondin-4, impacts synaptogenesis by promoting the formation of synapses at the presynaptic terminal. Cav α_2_δ-1 and thrombospondin-4 upregulation was reported in DRG and spinal dorsal horn neurons in NP mice models. Gabapentin inhibits the interaction of Cavα_2_δ-1 and thrombospondin-4, resulting in decreased cellular calcium influx, inhibiting neurotransmission, and exerting analgesic effects on sensory neurons in the spinal dorsal horn at the post-synaptic terminal [52]. Moreover, the association of α_2_δ with N-methyl-D-aspartate (NMDA) receptors has opened up new avenues in chronic pain. We know that nerve injury increases α_2_δ-1, intensifying pain signals through spinal cord NMDA receptors. A recent study demonstrated that nerve injury alters histones, reducing histone deacetylase-2 (HDAC2) in the *Cacna2d1* gene promoter in DRG neurons. HDAC2 removal induces lasting pain hypersensitivity, which is reversible via the use of NMDA blockers or gabapentin. Reduced HDAC2 heightens α_2_δ-1 and NMDA activity in the spinal cord; *Cacna2d1* knockout mice have milder pain responses. HDAC2 limits chronic pain by curbing α_2_δ-1. This insight challenges traditional HDAC roles. Restoring HDAC2 function or reducing histone acetylation could offer lasting nerve pain relief [53].

Another study featuring *Cacna2d1* knockout mice reported the significant attenuation of mechanical and cold allodynia in response to sciatic nerve injury when compared with wild-type mice. The Cav α_2_δ-2 subunit is known to limit axon growth via calcium influx through calcium channels in the PNS. Additionally, *Cacna2d2* gene ablation in vitro or pharmacological inhibition by pregabalin in vivo has been shown to facilitate axon regeneration after spinal cord injury [51]. In comparison, the deletion of the *Cacna2d3* gene in mice models has been associated with analgesia and the obstruction of the somatosensory system (triggered by thermal pain) [51,54]. However, CACNA2D4 variants have not been linked to NP [51].

### 2.2. Potassium (K+) Channels

Potassium (K+) channels are the most diverse ion channel family, with approximately 90 K+ channels being distinguished by their activation mechanisms and structures. The four major subgroups are as follows: (1) voltage-gated (K_V_), (2) calcium-activated (K_Ca_), (3) two-/tandem-pore domain (K_2P_), and (4) inwardly rectifying (K_IR_) [55,56]. In addition to establishing the resting membrane potential, K+ channels govern neuronal excitability. This is especially important in axons, where K+ currents assist in governing neuronal firing and modulating the action potential by counteracting the depolarization caused by the other excitatory channels present in axons. Furthermore, the intracellular location of potassium channels at neuron terminals suggests that they might affect cell–cell communication in reward circuitries through dopamine (DA) neurotransmission [56]. This review will further elaborate on potassium channel genes that contribute to painful neuropathies.

#### 2.2.1. KCNA1 and KCNA2

Currents of K_v_ channels have been detected in peripheral neurons (including DRG) and linked to pain pathways [45]. A decreased density of K_v_ channels has been observed in multiple pain conditions, such as nerve injury, painful diabetic neuropathy, and inflammation [57,58]. Here, we focus on K_v_1.1 and K_v_1.2, which are encoded by the *KCNA1* and *KCNA2* genes, respectively. Several animal models have been utilized to understand their function in pain pathophysiology [23,59,60]. In one study, knockout *kcna1* mice exhibited hyperalgesia in several behavioral tests, including the paw flick assay, hot plate assay, and formalin-induced hind paw licking [59]. The downregulation of *KCNA2* has been observed in the DRG of spinal nerve ligation rats, while *KCNA2* over-expression has been shown to diminish injury-induced pain hypersensitivity in rats [23]. Consistent with this, long noncoding *kcna2* antisense RNA silenced *KCNA2* and contributed to NP in rats via reducing the voltage-gated potassium current and increasing DRG excitability [61]. In addition, several studies have linked the epigenetic repression of KCNA2 with the development of NP [62,63,64,65,66,67]. Based on the above, K channels could be an appropriate target for pain therapy in specific individuals.

#### 2.2.2. KCNQ2/3/5

KCNQ or K_v_7 channels are responsible for the generation of M currents (I_M_) that control membrane potential and neuronal excitability in the central and peripheral nervous system [68]. It has been demonstrated that *KCNQ2*/*3*/*5* are present in rat nociceptive DRG neurons, and the expression of these three genes has been investigated specifically in NP diabetic rats. In one study, the tested animals had significantly decreased *KCNQ2*/*3*/*5* mRNA and protein levels, followed by a reduction in I_M_ and increased neuronal excitability of DRG. Moreover, mechanical allodynia and thermal hyperalgesia in diabetic rats was attenuated after KCNQ channel activation with retigabine, while the application of a KCNQ inhibitor, XE991, enhanced pain behaviors [69]. A similar effect was observed in another study on orofacial NP rats with downregulated *KCNQ2*, as they exhibited mechanical allodynia, which was alleviated after retigabine administration [70]. Interestingly, GOF mutations of *KCNQ2* and *KCNQ3* confer pain resilience via an effect on peripheral sensory neurons in inherited erythromelalgia individuals with a disease-causing Na_v_1.7 variant while *KCNQ3* genetic variants have been also linked to SFN (Table 1) [71,72].

#### 2.2.3. KCNS1 (K_v_9.1)

Potassium voltage-gated channel subfamily S member 1 (*KCNS1*), also recognized as K_v_9.1, is the only member of the K_v_S family with well-documented implications in pain states [57]. A single-nucleotide polymorphism of *KCNS1* has been associated with an increased risk of developing NP, although this does not confer causality (Table 1) [19]. *KCNS1* is highly expressed in neuronal tissues, including the DRG, spinal cord, and brain [73]. It has been shown that the mRNA of *KCNS1* is downregulated after neve injury in sensory neurons, while K_v_9.1 knock-down via siRNA injection has been shown to lead to neuropathic pain behaviors in tested rats [74]. These results were consistent with that of a later study involving transgenic mice lacking *KCNS1* in sensory neurons. The deletion of *KCNS1* increased basal mechanical pain, and after neuropathic injury, the knockout animals exhibited cold and mechanical hypersensitivity [75].

### 2.3. Hyperpolarization-Activated Cyclic Nucleotide-Gated (HCN) Channels

Hyperpolarization-activated and cyclic nucleotide-gated (HCN) channels belong to a gene-family consisting of four isoforms (HCN1-4) [76]. HCNs are known to form integral transmembrane proteins that act as voltage-gated cation channels that conduct both Na^+^ and K^+^ [77]. Members of the HCN family are expressed in different tissues, including the brain, heart, and peripheral neurons, where they are responsible for the generation of cation currents (I_f_ or I_h_). Their action is directly regulated by cyclic nucleotides, mainly cyclic adenosine monophosphate (cAMP), which contributes to the pacemaker activity in cardiac cells and neurons [77,78]. Several studies involving animal models have revealed the role of I_h_ in NP pathogenesis and pain processing [79]. Moreover, dysfunction in HCN channels, especially HCN1, HCN2, and HCN4 has been linked to multiple neurological and neurodegenerative disorders, including epilepsy, Alzheimer’s disease, Parkinson’s disease, and NP [80].

#### 2.3.1. HCN1

Among the HCN isoforms expressed in DRG, HCN1 is the most abundant, and it has been detected in all subtypes of sensory neurons, predominantly in large and medium-sized DRG neurons [81]. Several reports have highlighted that HCN current upregulation is closely linked to pathological pain condition [79,82,83]. In particular, the HCN1 role in NP has been investigated using a variety of animal models [84,85,86]. A significant upregulation of HCN1 pacemaker currents in large-diameter DRG was observed in a spinal nerve ligation (SNL) rat model. The increased density of I_h_ was accompanied by mechanical allodynia, which was reversed in the SNL rats after the administration of ZD7288, an HCN antagonist [82]. The contribution of I_h_ to tactile allodynia was also confirmed in chronic constriction nerve injury (CCI) and chronic compression-induced nerve injury rats [87,88]. Moreover, in the second model, hyperalgesia was also observed [87]. In addition to rat studies, in one particular publication, a *HCN1*^−/−^ mouse model was generated to assess the effect of HCN1 deletion on inflammatory hyperalgesia and NP. The HCN1 knockout mice were characterized by ablated I_h_, especially in large sensory neurons, and they exhibited decreased cold allodynia compared to wild-type animals [85]. Many strategies have been applied to target HCN1, providing more insight into the possible mechanism of action and potential pain therapy. In addition to the non-specific HCN blocker ZD7288, other components have also been tested [89,90]. Many of these drugs are known to be effective to reverse neuropathy but often cause side effects such as bradycardia [89,91]. Resta et al. reported upregulated I_h_ current in DRG neurons from a oxaliplatin-induced NP rat model. The selective HCN1 inhibitor MEL57A has been tested and shown to be effective in reducing hyperalgesia and allodynia in neuropathy rats without unwanted cardiac effects [91]. Taken together, these results indicate that HCN1 is a critical component of NP and that it could be specifically targeted to ameliorate pain symptoms.

#### 2.3.2. HCN2

HCN2 is a cAMP-sensitive isoform that has been intensively investigated, especially with respect to NP pathogenesis, often together with cAMP-insensitive HCN1 [84,86,91]. HCN2 is known to colocalize with HCN1 in large and medium-sized DRG neurons [81]. However, its expression in large sensory neurons is lower than HCN1 as HCN2 generally predominates in medium-sized/small DRG neurons [79]. Taking into account that many HCN blockers are nonspecific, the genetic deletion of HCN2 seems to be a valuable tool to understand its function. In one study, global HCN2 deletion in mice resulted in signs of epilepsy, ataxia, and premature death; therefore, mice with selectively ablated *HCN2* in nociceptive neurons expressing Na_V_1.8 were created to study its pathophysiology [92,93].

Na_V_1.8-*HCN2*^–/–^ mice display no behavioral manifestation of NP in response to both thermal and mechanical stimuli after nerve injury, according to the authors of [93]. Interestingly, in another study, HCN2 deletion from nociceptors decreased mechanical allodynia but not heat hyperalgesia in inflammatory chronic pain mouse models [83]. Tsantoulas et al. investigated HCN2’s role in diabetic neuropathy pain using type 1 and 2 diabetes mice. They found an increased level of cAMP in the somatosensory neurons of the tested animals and proposed that this is the reason why excessive HCN2 activation leads to the firing of repetitive nociceptive nerve fibers and associated effects in diabetic neuropathy pain. In addition to that, it was shown that mechanical allodynia in painful diabetic mice can be reverted via the administration of the HCN blocker ivabradine and HCN2 deletion in small nociceptive neurons [94]. Recently, many researchers’ efforts have been dedicated to understanding the contribution of HCN2 to NP states. It has been found that HCN2 induces NP through the upregulation of NR2B and the activation of the CaMKII/CREB cascade in spinal neurons in oxaliplatin-induced NP [95]. Furthermore, decreased HCN2 expression inhibits pro-inflammatory reactions and suppresses nuclear factor NF-κB activation, which is involved in NP progression [96].

#### 2.3.3. HCN3 and HCN4

HCN3 and HCN4 subunits are expressed in primary sensory neurons; however, their expression level in DRG neurons is lower in comparison to HCN1/2 [79,81]. Although HCN3 was found to be expressed in small, medium, and large sensory neurons, it seems that its role in acute and chronic pain is limited. A study performed on HCN3 knockout mice with nerve damage did not show differences in thermal hyperalgesia in comparison to wild-type animals. *HCN3* gene deletion also did not result in significant changes in mechanical hyperalgesia; however, slightly reduced responses in the pinprick test were observed. Overall, these results indicate that HCN3 is not a major component contributing to NP [97]. HCN4 is known to be expressed in the brain and DRG, predominantly in medium and small sensory neurons [80]. The role of HCN4 in the pathogenesis of epilepsy has been well established, as several pathogenic mutations have been identified [98,99]; nevertheless, this kind of evidence is missing for the NP phenotype.

### 2.4. Anoctamin Gene Family

The Anoctamin protein family consists of 10 members (ANO1-10). Anoctamins, also known as TMEM16, are involved in ion transport, phospholipid scrambling, and membrane protein regulation [100]. ANO1 (TMEM16A) and ANO3 (TMEM16C), classified as Ca^2+^-activated Cl^-^ channels (CaCCs), are known to play a crucial role in pain processing in sensory neurons [101,102]. Both channels are expressed in nociceptive DRG neurons [100,103]

#### 2.4.1. ANO1

ANO1 is a ligand-gated anion channel that is activated as a result of calcium entering through TRPV1 [102]. ANO1 serves as a heat sensor in somatosensory neurons activated by noxious temperatures >44 °C in the absence of intracellular Ca^2+^. ANO1 activity in DRG is associated with pain hypersensitivity in inflammatory and NP animal models [104]. Conditional *ANO1* knockout mice have showed reduced thermal nociceptive responses [103,105]. Furthermore, *ANO1* knockout mice have exhibited significantly reduced inflammatory hyperalgesia and mechanical allodynia [104]. These findings are consistent with a previous study on bradykinin-induced inflammatory pain rats, which demonstrated that inhibiting ANO1 results in pain attenuation [106]. The inhibition of TRPV1-ANO1 interaction by TRPV1 antagonists could be an alternative way to treat PPN (Table 1) [102]

#### 2.4.2. ANO3

ANO3 functions as a calcium-dependent phospholipid scramblase as ANO3 does not exhibit CaCC activity since it does not produce Cl- currents stimulated by intracellular Ca^2+^ [100]. In one study, the genetic ablation of ANO3 resulted in increased mechanical and thermal sensitivity in TMEM16C knockout mice. Electrophysiological studies have revealed an interaction between ANO3 and sodium-activated potassium Slack channels. In one study, ANO3 did not form an ion channel itself but enhanced the Slack channel’s activity in the DRG neurons and regulated pain processing [101]. Several Variants of Uncertain Significance (VUS) have been reported in SFN and painful DPN (Table 1).

### 2.5. Transient Receptor Potential (TRP) Cation Channels

The transient receptor potential channel is a super-family of genes known to be associated with nociception and pain perception. This family is divided into twenty-eight elements which are further segregated into six subfamilies found in mammals: ankyrin (TRPA), canonical (TRPC), melastatin (TRPM), mucolipin (TRPML), polycystin (TRPP), and vanilloid (TRPV). All the TRP genes are non-selective cation-permeable channels that are known to conduct calcium ions (Table 1) [107]. The TRPA, TRPM, and TRPV channels have been shown to be associated with PPN (Table 1).

#### 2.5.1. TRPA1

TRPA1 is a type of transient receptor potential cation channel found in various parts of the body, especially in nociceptive neurons responsible for sensing pain [107]. It serves as a chemosensor, reacting to chemical irritants and causing painful burning sensations. Additionally, it can be activated by cold stimuli, which sets it apart from the TRPM8 channel [108,109].

TRPA1 is coexpressed with TRPV1, influencing thermosensation and contributing to both pain and inflammation [110,111]. In a recent study, systematic therapy with Sigma-1 receptor (an endoplasmic reticulum chaperone) antagonists reduced painful symptoms in an oxaliplatin-induced neuropathy mice model through regulating TRPA1. Targeting TRPA1 with these antagonists has the potential to prevent and treat Chemotherapy-Induced Peripheral Neuropathy (CIPN) and other NP syndromes, opening the door to new pain management therapies [112]. A recent study discovered that TRPA1 in Schwann cells contributes to pain caused by CGRP and capsaicin in mice and rats. TRPA1 antagonists reduced pain in less severe neuropathic pain models and showed some effectiveness in patients with milder neuropathy. TRPA1 might have a role in neurogenic inflammation and moderate nerve injury-related pain, whereas CGRP does not seem to be involved in these conditions [113]. Another study suggested that the co-localized potassium Slack channel could modulate the TRPA1-mediated activation of sensory neurons but not TRPV1-mediated activation [114]. Moreover, a *trpv1-trpa1-trpm3* knockout mice model showed nearly no thermal sensitivity to nociception, indicating that a variety of TRP channel genes engage in thermal hyperalgesia [115].

Certain *TRPA1* genetic markers have been associated with specific characteristics, including central pain hypersensitivity, primary and secondary hyperalgesia, mechanical nociception, and cold hyperalgesia, following inflammation and nerve injury [108,116]. Genetic variants of the *TRPA1* gene have been linked to conditions like familial episodic pain syndrome (FEPS) and cram-fasciculation syndrome (CFS), as mentioned in Table 1 [108,117]. More recently, rare TRPA1 variants have been shown to be significantly enriched in chronic neuropathic and nociplastic pain patients [116].

#### 2.5.2. TRPM2

The structure of Transient Receptor Potential Cation Channel Subfamily Melastatin Member 2 (TRPM2) is similar to other TRPM channels, except for the presence of a nucleoside diphosphate-linked moiety x motif 9 (NUDT9) homologous motif at its C-terminal side [107]. When adenosine phosphate-ribose binds to this motif, it modifies the channel’s gating, enabling the influx of calcium and sodium ions. TRPM2 can be activated by various stimuli, such as adenine dinucleotide, reactive oxygen species (ROS), and intracellular calcium ions [118]. Studies in mice have indicated that TRPM2 expression in macrophages and spinal glial cells aggravates inflammatory signals associated with pain, influencing the pathophysiology of inflammation and NP [119,120,121,122]. However, the precise mechanism of TRPM2 in NP is not fully understood. A recent study investigated the potential of hesperidin (HES) to alleviate diabetic neuropathy (DNP) through TRPM2 channel modulation. HES treatment in diabetic rats lowered hyperglycemia, pain sensitivity, and nerve damage by controlling TRPM2 channel activity. This suggests that HES could potentially mitigate DNP by targeting the TRPM2 channel [123].

In addition to its role in pain, rare missense variants of TRPM2 have been associated with several other conditions, including bipolar disorder, amyotrophic lateral sclerosis, Parkinson’s disease, trigeminal neuralgia, and corneal neuralgia [22,24,124]. Table 1 shows the rare missense variants reported for trigeminal and corneal neuralgia.

#### 2.5.3. TRPM3

The transient receptor potential melastatin-3 (TRPM3) channel is present in various tissues, including the DRG and cardiac and pancreatic cells [125]. It can be activated by sphingosine, pregnenolone sulfate (a neuro-steroid), hypo-osmolality, and temperatures above 37 °C [125,126]. TRPM3 is involved in thermal hyperalgesia and insulin secretion in humans [127,128]. Studies in TRPM3 null mice showed the channel’s role in thermal hyperalgesia during inflammation [129,130]. The Gβγ subunit of heterotrimeric G proteins blocks TRPM3, leading to pain reduction and providing insights into the pain-relieving mechanisms of opioid analgesics [131].TRPM3 has VUS variants associated with developmental and epileptic encephalopathy and trigeminal neuralgia (shown in Table 1) [22,132]. As mentioned before, the ablation of the combination of these three genes (*trpa1*-*trpv1*-*trpm3*) in mice models contributed to thermal nociception. The inhibition of all three channels with a single drug or other therapeutic options could be a promising approach for alleviating thermal hyperalgesia [115]. Moreover, in mice model studies, TRPM3 channel activity has been shown to be increased after oxaliplatin treatment, while mice lacking TRPM3 supposedly do not experience cold and mechanical pain. Also, the intraperitoneal injection of isosakuranetin, a TRPM3 inhibitor, has been shown to reduce pain behavior in mice. Thus, the findings mentioned above indicate that TRPM3 could be a new target for oxaliplatin-related-CIPN [133].

#### 2.5.4. TRPM8

The Transient Receptor Potential Cation Channel Subfamily Melastatin Member 8 (TRPM8), also referred to as cold-menthol receptor 1, was among the initial channels identified as cold sensors [134]. TRPM8 is found in primary afferent nerve fibers of the Aδ and C types located in the DRG, as well as in the bladder and prostate [135,136,137]. As a non-specific cation channel, TRPM8 plays a role in regulating calcium ion balance. It responds to cold temperatures (8–28 °C), alterations in pH, and various cooling agents like methanol, icilin, and eucalyptol [134,138,139]. Cold hyperalgesia, a heightened sensitivity to cold, can be induced by chemotherapy in cancer patients. Mouse models with NP treated via chemotherapy drugs such as oxaliplatin and vincristine have been observed to display a significant rise in TRPM8 mRNA levels in DRG neurons. Consequently, either eliminating the TRPM8 gene or using drugs that counter TRPM8’s effects seemingly alleviates NP in these models [140,141]. However, a recent study suggested that after oxaliplatin or paclitaxel treatment, TRPM8-expressing trigeminal neurons exhibit higher vulnerability rather than DRG neurons [142]. Morphine-triggered cold hyperalgesia activates the μ-opioid receptor, leading to the excitation of DRG neurons that express TRPM8 [143]. Meanwhile, in another study, RGM8-51, a TRPM8 antagonist, was effective in decreasing cold hyperalgesia in an oxaliplatin-induced PN mouse model and relieved NP rats of cold, mechanical, and heat hypersensitivity after sciatic nerve constriction [144]. Another investigation explored Ajugarin-I (Aju-I) in diabetic NP. Aju-I treatment in rats reduced hyperglycemia, pain hypersensitivity, and pancreas damage induced by diabetes. It also mitigated histopathological changes in nerves and the spinal cord, decreasing oxidative stress and modulating TRPV1/TRPM8 nociceptors [145]. The only documented GOF VUS within *TRPM8*, rs200774365 (Arg30Gln), has been linked to trigeminal neuralgia [25].

#### 2.5.5. TRPV1

The Transient Receptor Potential cation channel Vanilloid 1 (TRPV1) is expressed in both somatic and visceral afferent neurons present at both spinal cord and peripheral junctions [146]. It functions as a polymodal nociceptor, responding to a diverse range of noxious stimuli such as vanilloids, protons, heat (≥42 °C), lipids, and voltage changes. Given its multi-faceted gating mechanisms, TRPV1 has garnered attention as a potential molecular target for various pain-related conditions, including hyperalgesia, inflammatory pain, hypersensitivity, and acute burning pain [13,146,147,148]. Over the past decade, significant efforts have been made to investigate the role of TRPV1 in pain modulation. Researchers have developed a variety of antagonists and genetic animal models to explore its function. However, while these antagonists have demonstrated efficacy in treating NP based on their modality, some have exhibited the unintended consequence of inducing hypo- or hyperthermia in animal models or patients [149]. Some of the recent animal model studies on rodent species have explored different types of therapeutic approaches, as outlined in Table 2. Studies involving TRPV1-null mice have provided valuable insights into its role. These mice display reduced sensitivity to thermal pain in response to inflammation, suggesting the involvement of TRPV1 in thermal hyperalgesia [150]. As indicated before, the trio TRP channel knockout model of *trpa1*-*trpv1*-*trpm3* has revealed that multiple TRP channel genes likely collaborate in orchestrating thermal sensations and nociception [115].

Considering these findings, TRPV1 emerges as an attractive molecular target, particularly for individuals suffering from peripheral polyneuropathy (PPN) and NP. Extensive pharmacological and genetic research has focused on modulating TRPV1’s activity. Despite these efforts, the genetic landscape of rare functional variants contributing to or causing NP remains largely unexplored. However, from their studies on NP, Ślęczkowska et al. and Katz et al. obtained potentially causal and pathogenic variants, as listed in Table 1 [9,151]. It is worth noting that Katz et al. highlighted a rare homozygous TRPV1 functional variant that renders the channel completely non-functional, resulting in reduced heat sensitivity and heightened cold sensitivity [151].

**Table 2:** Summary of recent rodent studies on the therapeutic use of TRPV1-related mechanisms in various pain conditions.

Disease/Condition	Therapeutic Approach	References
CIPN	TRPV1 siRNA into a druggable approach	[152]
PDPN	Potential target blocking GPR177-WNT5a-TRPV1 axis	[153]
PDPN	Alpha-lipoic acid and capsazepine together inhibits TRPV1 channel	[154]
PDPN	Ajugarin-I treatment reduces TRPV1/TRPM8 expression	[145]
NP	Pregablin reduces pain perception via PKCε/TRPV1 pathway	[155]
Side-effects of opioids post-NP	Potential target such as β-arrestin 2 that regulates bi-directionally TRPV1 and μ-opioid receptors	[156]
NP	Cannabidiol for acute NP partially inhibits 5-HT1A and TRPV1	[157]

#### 2.5.6. TRPV3

TRPV3 (Transient Receptor Potential cation channel Vanilloid 3) is highly expressed in skin and hair follicle keratinocytes, as well as in DRG [158]. TRPV3 is involved in cutaneal development and maintenance and results in an itchy sensation [159]. TRPV3 is activated at a mild temperature of ~33 °C [160]. Unlike other TRP channels, TRPV3 becomes sensitized after repeated stimulation rather than desensitization [161]. The heat-induced activation of TRPV3 leads to increased channel expression and releases a substance called thymic stromal lymphopoietin, which is a potent itch inducer [162]. Moreover, GOF variants in *TRPV3* have been associated with severe pain, along with itchiness [162], and VUS has been associated with several different pain disorders, including SFN (Table 1) [9,26,163]. Additionally, citrusinine-II, a plant-derived medication, is known to block the TRPV3 channel to relieve pain and itchiness (Table 1) [164].

#### 2.5.7. TRPV4

TRPV4, also known as Osmosensitive Transient Receptor Potential Channel 4, is similar to TRPV2 and found in sensory neurons, non-neuronal cells, and keratinocytes. TRPV4 is activated by mild warm temperatures (~30–35 °C) and hypo-osmolarity, much like TRPV3 [159,165]. Todaka et al. discovered that mice lacking TRPV4 exhibit changes in sensory peripheral neurons when exposed to hypo-osmolarity and mechanical nociception [166]. The TRPV4 gene is extensively expressed in the brain and spinal cord, including regions like the DRG, TG, nodose ganglion, and hypothalamic neurons [167]. According to Bagnell et al., TRPV4, along with the small GTPase RhoA, forms a sensitive signaling complex that contributes to detrimental changes in cell structure during neurological injuries and diseases. Thus, they suggest that targeting TRPV4 with drugs could prevent these harmful effects, offering new possibilities for treating neurological conditions that currently lack effective treatments [168]. The authors of another study drawn a similar conclusion, stating that after nerve injury, TRPV4 activation causes mechanical pain and triggers TRPV4-dependent microgliosis. Moreover, TRPV4’s involvement in increasing neuronal excitability, dendritic spine remodeling, and spinal neuroplasticity through microglia-derived lipocalin-2 has been noted in the literature, thereby making the channel a more plausible target for NP [169]. Pathological TRPV4 variants can lead to ion channel dysfunction, resulting in motor issues and axonal neuropathies such as spinal muscular atrophy, distal hereditary motor neuropathy, and Charcot–Marie–Tooth disease 2C [170]. The pathological TRPV4 variants mentioned in the literature are detailed in Table 1.

### 2.6. Acid-Sensing Ion Channels (ASICs)

ASICs are a family of four voltage-independent amiloride-sensitive cationic channels (ASIC-4) that are permeable to cations, primarily Na^+^. Extracellular protons activate ASICs, and they are abundant in the central nervous system and peripheral nerves. Several studies have suggested that ASIC1-3 plays a role in the pathophysiology of chronic, inflammatory, and neuropathic pain. In one study, an increased expression of ASIC1a, ASIC2, and ASIC3 mRNA was observed in DRG after inflammation [171]. However, in ASIC1^−/−^ and ASIC2^−/−^ mice, a lack of ASIC expression was associated with increased pain behavior, primarily during formalin injection [172]. Blocking ASIC1a with PcTx1 or specific antisense oligonucleotides has been shown to reduce thermal and mechanical hypersensitivity in CFA animals [173]. Citric acid stimulates and potentiates ASIC1, resulting in nociceptive responses induced by subcutaneous acid infusion [174]. Chen et al. found that ASIC3^−/−^ knockout mice were more sensitive to moderate- to high-intensity pain stimuli. However, mice lacking ASIC3 were hypertensive to high-intensity thermal stimulation but did not exhibit increased pain behaviors after formalin injection [175]. ASIC3 activation via acid and pruritogen has been shown to mediate itchiness, which is a common symptom of NP [176].

## 3. Conclusions

With a primary focus on ion channels, the field of neuropathic pain genetics is continuously expanding, leading to the discovery of new genes and variants. This provides opportunities to link genetic data with mechanistic studies and patient characteristics. This paper aims to integrate this information to optimize therapeutic approaches and identify novel treatment possibilities. Most of the ion channel genes discussed in this review are associated with hyperalgesia or allodynia, except for HCN3, which is not linked to pain, and ANO3 and HCN4, which indirectly impact pain perception. Variants in ion channel genes are also associated with specific clinical manifestations of NP and PPN, such as cold-induced pain (TRPA1 and TRPM8), inflammatory pain (CACNA1A, ANO1, TRPV1, TRPM2, and ASIC3), itch-related pain (ASIC3 and TRPV3), and chemotherapy-induced pain (CACNA1H, TRPV2, and TRPM8). This genetic complexity makes pain treatment challenging, as generalized approaches may not be very effective. Targeting multiple ion channels with drugs might offer a more suitable solution for a broader range of patients. For instance, drugs targeting TRPV1 can impact various underlying causes due to its interactions with several ion channels. However, it is important to note that TRPV1-targeting drugs tend to attenuate rather than completely reverse pain responses. Thus, developing drugs that target multiple ion channels for pain relief could prove more effective. Given the observed genetic heterogeneity in PPN patients, personalized medical treatment based on each patient’s genetic and clinical profiles may be more beneficial. This individualized approach aims to address specific clinical features, leading to an increased treatment response.

## Figures and Tables

**Table 1:** Genetic variants with their known phenotypic and functional effect related to neuropathic pain.

Gene	Mutation	Type of Evidence	Functional Effect	Phenotype	Reference
*ANO3*	Ser213Phe	Targeted sequencing	N/A	PDPN	[8]
	Ile453Val	Targeted sequencing	N/A	PDPN	[8]
	Leu984Phe	Targeted sequencing	N/A	PDPN	[8]
	Gly1034Arg	Targeted sequencing	N/A	SFN	[9]
	Met370Cysfs*?	Targeted sequencing	N/A	SFN	[9]
*CACNA1A*	Pro2455His	WCV patch clamp	GOF	TN	[17]
*CACNA1H*	Arg481Cys	WES	N/A	Writer’s cramp and TN	[18]
*HCN1*	Arg405Gln	Targeted sequencing	N/A	PDPN	[8]
*KCNS1*	Ile48Val	SNPs association with greater pain outcome	N/A	Pain after nerve injury	[19]
*KCNQ3*	Val629Ile	Targeted sequencing	N/A	SFN	[9]
	Asp569Gly	Targeted sequencing	N/A	SFN	[9]
*TRPA1*	Ser86Ala	WCV patch clamp and calcium imaging	GOF	N/A	[20]
	Leu118Val	Targeted sequencing	N/A	PDPN	[8]
	Thr311Asn	Targeted sequencing	N/A	SFN	[9]
	Ser317Ala	WCV patch clamp and calcium imaging	GOF	N/A	[20]
	Tyr327Cys	Targeted sequencing	N/A	SFN	[9]
	Arg343Cys	Targeted sequencing and Sanger sequencing	N/A	EM	[21]
	Arg393Gln	WES	N/A	TN	[22]
	Arg393*	Targeted sequencing	N/A	SFN	[9]
	Ser428Ala	WCV patch clamp and calcium imaging	GOF	N/A	[20]
	Asn460Ser	Targeted sequencing and Sanger sequencing	N/A	EM	[21]
	Cys608*	Targeted sequencing and Sanger sequencing	N/A	EM	[21]
	Arg652*	Targeted sequencing	N/A	PDPN, SFN	[8,9]
	Met689Val	Targeted sequencing	N/A	SFN	[9]
	Ala828Leufs*17	Targeted sequencing	N/A	PDPN	[8]
	Asn855Ser	WCV patch clamp	GOF	Episodic pain syndrome	[23]
	Ser972Ala	WCV patch clamp and calcium imaging	GOF	N/A	[20]
	Lys1046Glu	Targeted sequencing	N/A	SFN	[9]
	Ser443Gly	WES	N/A	TN	[22]
*TRPM2*	Asp624Trp	NGS study	N/A	CN	[24]
	Ala890Val	NGS study	N/A	CN	[24]
	Val934Ile	NGS study	N/A	CN	[24]
	Ala1645Val	WES	N/A	TN	[22]
*TRPM3*	Arg30Gln	WCV patch clamp and calcium ion imaging	GOF	TN	[22,25]
*TRPM8*	Asn222Ser	Targeted sequencing	N/A	SFN	[9]
	Arg368Trp	Targeted sequencing	N/A	SFN	[9]
	Glu479Asp	Targeted sequencing	N/A	PDPN	[8]
	Asp665Asn	NGS study	N/A	CN	[24]
	Val705Glyfs*79	Targeted sequencing	N/A	PDPN	[8]
	Thr732Ile	Targeted sequencing	N/A	PDPN	[8]
	Val915Met	NGS study	N/A	CN	[24]
	Thr982Met	Targeted sequencing	N/A	SFN	[9]
	Gln85Arg	Whole-cell voltage clamp	GOF	CN after refractive surgery	[24]
*TRPV1*	Phe305Ser	Targeted sequencing and Sanger sequencing	N/A	EM	[21]
	Phe305Cys	Targeted sequencing	N/A	SFN	[9]
	Thr450Ala	Targeted sequencing	N/A	SFN	[9]
	Gln498*	Targeted sequencing and Sanger sequencing	N/A	EM	[21]
	Arg579Cys	Targeted sequencing	N/A	SFN	[9]
	Gly568Asp	Case report	N/A	Painful focal plantar keratoderma	[26]
*TRPV3*	Gly568Cys	NGS study and WCV patch clamp	GOF	OSLP and EM	[26]
	Gly568Val	Sanger sequencing	N/A	OSLP	[26]
	Gly573Ser	Animal model study	GOF	Severe itching	[27]
	Arg416Trp	Sanger sequencing	N/A	OSLP	[26]
	Arg416Gln	NGS study and WCV patch clamp	indirectly involved in channel gating	OSLP	[26]
	Leu673Phe	Sanger sequencing	moderately affects channel function	Atypical OSLP	[26]
	Trp692Ser	Sanger sequencing	severely affects channel function	OSLP	[26]
	Leu669Pro	Targeted sequencing	N/A	SFN	[9]
	p.? #	Targeted sequencing	N/A	SFN	[9]
	Arg186Gln	NGS study and qRT-PCR	N/A	HMSN2C and HMN8	[28,29]
*TRPV4*	Arg232Cys	NGS study and qRT-PCR	N/A	HMSN2C and HMN8	[30]
	Arg269Cys	NGS study and qRT-PCR	N/A	CMT2C	[28]
	Arg269His	NGS study and qRT-PCR	GOF	CMT2C	[28,30]
	Tyr283Asn	WES	N/A	TN	[22]
	Arg316Trp	NGS study and WCV patch clamp	GOF	HMSN2C	[31,32]
	Arg316His	NGS study and WCV patch clamp	GOF	HMSN2C	[30]

GOF, gain-of-function; N/A, not applicable; SNP, single-nucleotide polymorphism; GWAS, genome-wide association studies; NGS, next-generation sequencing; WES, whole exome sequencing; qRT-PCR, real-time quantitative reverse transcription PCR; WCV, whole-cell voltage; PDPN, painful diabetic peripheral neuropathy; SFN, small-fiber neuropathy; EM, erythromelalgia; TN, trigeminal neuralgia; CN, corneal neuralgia; OSLP, Olmsted syndrome with lesional pain; HMSN2C, hereditary motor and sensory neuropathy 2C; HMN8, distal hereditary motor neuronopathy type 8; CMT2, Charcot–Marie–Tooth type 2 disease. ^#^ changed protein length due to splicing event at position c.1242+1G>A (loss of donor splice site of intron 9).

## Data Availability

Not applicable.
